# Screening of Allelochemicals in *Miscanthus sacchariflorus* Extracts and Assessment of Their Effects on Germination and Seedling Growth of Common Weeds

**DOI:** 10.3390/plants9101313

**Published:** 2020-10-05

**Authors:** Bimal Kumar Ghimire, Myeong Ha Hwang, Erik J. Sacks, Chang Yeon Yu, Seung Hyun Kim, Ill Min Chung

**Affiliations:** 1 Department of Crop Science, College of Sanghuh Life Science, Konkuk University, Seoul 05029, Korea; bimal_g12@yahoo.com (B.K.G.); kshkim@konkuk.ac.kr (S.H.K.); 2 Bioherb Research Institute, Kangwon National University, Chuncheon 24341, Korea; mwung.ha5471@daum.net (M.H.H.); cyyu@kangwon.ac.kr (C.Y.Y.); 3 Department of Crop Science, University of Illinois, Urban-Champaign, 1201 W, Gregory Dr., Urbana, IL 61801, USA; esacks@illinois.edu

**Keywords:** *Miscanthus sacchariflorus*, allelopathy, germination, phenolic compounds, antioxidant enzyme

## Abstract

There is increasing interest in the application of bioherbicides because they are less destructive to the global ecosystem than synthetic herbicides. Research has focused on reducing the dependence upon synthetic herbicides by substituting them with environmentally and economically sustainable bioproducts. Allelopathic phytochemicals may be an efficient method for controlling weeds, benefitting both the environment and human health. This study addressed the allelopathic potential of *Miscanthus sacchariflorus* (MS) extracts on the germination, plant growth, biomass, and biochemical parameters (electrolyte leakage, photosynthetic pigments, and antioxidant enzyme activities) of weeds using laboratory and field experiments. Liquid chromatography-mass spectrometry/mass spectrometry (LC-MS/MS) showed the presence of 22 phenolic compounds, including Orientin, Luteolin, Veratric acid, Chlorogenic acid, Protocatechuic acid, *p*-Coumaric acid, and Ferulic acid. Leaf extracts of *M. sacchariflorus* either completely suppressed or partially reduced seed germination and affected the development of weed seedlings (root and shoot length), in a dose-dependent manner. Aqueous extracts of *M. sacchariflorus* reduced the fresh weight and dry weight, affected the photosynthetic pigment content (chlorophylls, carotenoids), influenced the electrolyte ion leakage, and stimulated the activity of antioxidant enzymes in a species-specific manner. Pearson’s correlation analysis showed that the phenolic compound composition of *M. sacchariflorus* correlated with the variables tested, indicating that the phytochemicals present in the plant extracts of *M. sacchariflorus* are a potential source of bio-herbicides.

## 1. Introduction

*Miscanthus sacchariflorus* (Maxim.) Hack, a perennial herbaceous plant belonging to the Poaceae family, has been recognized as a potential bioenergy crop [1,2,3]. *M. sacchariflorus* grows primarily in the temperate regions of Asia and eastern Russia [4,5] at various altitudes, though not above 2000 m [3,6,7,8]. Recently, *M. sacchariflorus* has been indicated as a possible bioenergy crop due to its potential to outperform other bioenergy crops with regard to annual biomass production and its ability to grow in a wide range of environments with minimal inputs [2,3,7,8,9]. *M. sacchariflorus* competes poorly with weeds during its initial years of establishment, with significant negative effects to its growth and biomass due to deprivation of nutrients, space, light, and moisture [10]. Comparatively, most weeds have excellent adaptive features and can germinate in diverse climatic conditions [11]. Most of these weeds have a shorter dormancy period and prolific seed production, producing intense competition for resources with crops in cultivated fields. For example, *Bidens frondosa* and *Digitaria sp*. produce a large quantity of seeds, which are easily dispersed over large distances and possess the potential to germinate in diverse climatic conditions [12,13,14]. *Chenopodium album*, a fast-growing broad-leaved weed, has been reported to be relatively insensitive or resistant to herbicides such as triazine [15,16]. Barnyard grass, another vigorously growing weed, can produce over one million viable seeds per plant [17,18] and is resistant to multiple herbicides including chloroacetamide (butachlor) and acetanilide (propanil); propagation of this grass causes significant losses in crop yields every year [19,20]. Moreover, a number of previous studies have reported that the allelochemicals released by these weeds to the surrounding environment can directly or indirectly inhibit the biomass production and yield of cultivated crops [18,21]. For example, *Erigeron canndensis* presents strong competition to food crops in terms of growth and also produces phytotoxic chemicals that can suppress the growth of cultivated crops [22,23,24,25]. Earlier studies have indicated that the allelopathic properties of weeds play an important role in the invasive and adaptative capacities of perennial broad-leaved and grass weeds in cultivated fields [26]. Miscanthus is considered as an invasive bio energy crops [27]. Invasive plants produce phytochemical with strong allelopathic properties to suppress co-occurring plant species [28,29]. However, the effects of *M. sacchariflorus* on weed germination have not been documented yet.

Indiscriminate use of herbicides for the control of weeds in order to increase cultivated plant growth and food production has yielded negative effects to non-targeted organisms, including human beings [30]. Consumption of potentially toxic chemical residues can trigger a wide range of effects on human health, including cancers, genetic disorders, endocrine disruption, and neurological problems [31]. Moreover, some studies have indicated that the improper and excessive use of herbicides is causing contamination of soil, air, water, and food resources. Herbicides used in the field can contaminate water resources due to soil runoff or leaching and cause genetic damage and physiological effects [32,33,34,35,36]. Mechanical weed control, on the other hand, is not only damaging to the root systems of cultivated plants, but also labor-intensive and time-consuming. Novel methods of controlling weeds are therefore, urgently needed to meet the demands of modern agriculture. As compared to conventional herbicides, those derived from natural products or their analogs have the potential to be safer and more environmentally friendly.

Many plant species produce phytochemicals that enable them to inhibit or suppress the germination or growth of other plants [37,38]. These biochemicals are also called allelochemicals and generally constitute phenolic compounds, saponins, terpenes, steroids, alkaloids, and quinones [39]. These compounds accumulate in the soil through plant residual decomposition, root exudation, and shoot leaching processes [40,41,42]. Various studies have reported the isolation of single allelopathic compounds and their application for the development of herbicides [43,44,45,46]. Moreover, a number of allelochemicals from potentially allelopathic plants species have already been isolated and have proven to successfully inhibit weed germination and growth [47], making them environmentally friendly compared to chemical herbicides [48]. Most of these allelochemicals are beneficial to the soil, as they improve the nutrient concentration and enhance the microbial activity of the soil [49,50]. Moreover, most of these allelo-compounds are partially or completely soluble in water, which simplifies their application even in the absence of surfactants [51]. Dayan et al. [52] argued that the absence of halogenated substitutes, the lower concentrations of allelopathins necessary for activity, and the higher specific properties due to diversity in the allelopathins make these compounds more environmentally friendly than chemical herbicides [53]. Accordingly, scientific interest in controlling weeds through allelochemicals has recently increased. Studies have reported that the allelochemical components of some plant species effectively inhibit the germination and growth of weeds, which are effects primarily attributed to the phenolic compounds present in the sample extracts [47,54]. However, no study has yet been conducted to evaluate allelopathic weed management systems for *M. sacchariflorus* under field conditions.

Therefore, the primary objective of the present study was to investigate the possible allelopathic effects of *M. sacchariflorus* extracts on the seed germination and growth of selected weed species. Further, we documented the major allelochemicals present in *M. sacchariflorus* leaf extracts using liquid chromatography-mass spectrometry/mass spectrometry (LC-MS/MS).

## 2. Results

### 2.1. LC-MS/MS Analysis of Phenolic Compounds in MS

**Figure 1:**
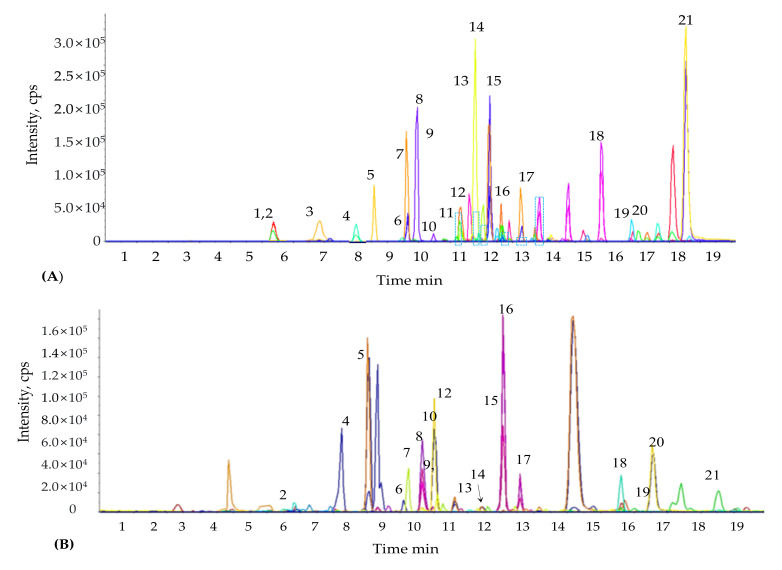
(**A**). Representative multiple reaction monitoring (MRM) ion chromatogram of phenolic compound standards, (**B**) Representative MRM ion chromatogram of phenolic compounds obtained from *M. sacchariflorus.* 1. L-Phenylalanine; 2, Gallic acid; 3, Homogentisic acid; 4,Protocatechuic acid; 5, Chlorogenic acid; 6, Orientin; 7, Rutin; 8, *p*-Hydroxybenzoic acid; 9, Caffeic acid; 10, Vitexin; 11, Vanillic acid; 12, Gentisic acid; 13, 2,4-dihydroxybenzoic acid; 14, *p*-Coumaric acid; 15, Ferulic acid; 16, *m*-Coumaric acid; 17, Veratric acid; 18, Luteolin; 19, Quercetin; 20, Salicylic acid; 21, Apigenin.

To analyze the possible relationships between allelopathic properties and the phytochemical constituents of the plant, the phenolic compounds of MS were quantified using LC-MS/MS (Table 1 and Table 2). Isolation and estimation of individual phenolic compounds were performed by comparing the retention time with standard reference compounds (Figure 1A,B). Phenolic compounds such as orientin, luteolin, veratric acid, chlorogenic acid, protocatechuic acid, *p*-coumaric acid, and ferulic acid were the most dominant phenolic acids and were detected in relatively higher concentrations. Among the dominant phenolic compounds, orientin and luteolin (512.71 ± 3.09 and 409.33 ± 3.32 µg g^−1^, respectively) were the most abundant flavones, while veratric acid and chlorogenic acid (384.38 ± 5.14 and 296.00 ± 1.00 µg g^−1^, respectively) were the predominant phenolics. Salicylic acid was the least abundant phenolic acid (1.24 ± 0.22 µg g^−1^) detected.

### 2.2. Effect of M. sacchariflorus Leaf Extracts on Seed Germination, Shoot Length, and Root Length of Weeds

**Figure 2:**
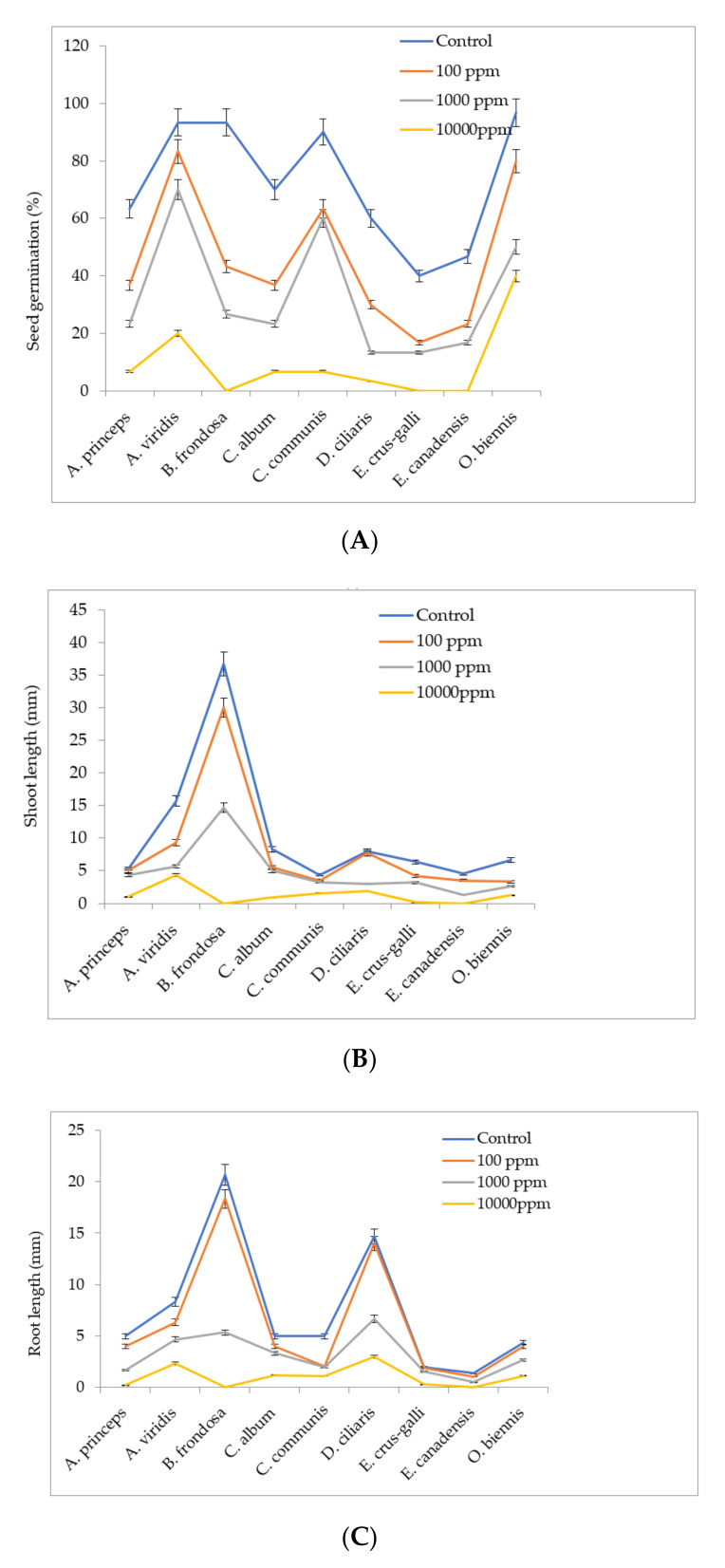
Effect of aqueous extract of M. sacchariflorus on (**A**) seed germination (%), (**B**) shoot length (mm), (**C**) root length (mm). Values are reported as mean ± standard deviation of three parallel experiments.

The allelopathic effects of leaf extracts of MS were investigated to determine its natural herbicidal effects against broadleaf (*Chenopodium album, Bidens frondosa, Amaranthus viridis, Artemisia princeps var. orientalis, Commelina communis, Oenothera biennis Erigeron canadensis*) and grass (*Digitaria ciliaris, Echinochloa crus-galli,*) weeds that typically grow in the studied fields. In the present study, the seed germination performance of all of the studied weeds was significantly affected by all of the treatments (Figure 2A). As indicated in the results, leaf extracts of *M. sacchariflorus* either completely suppressed or partially reduced the weed seed germination, although the effect was dependent upon the extract concentration. The germination of the weed seeds was most affected at higher concentration of leaf extracts of *M. sacchariflorus*. At the highest concentration of leaf extracts (10,000 ppm), the germination of weeds seeds was reduced by 100% in *Bidens frondosa, Echinochloa crus-galli,* and *Erigeron Canadensis* L. Comparatively, *Oenothera biennis* and *Amaranthus viridis* seeds were least sensitive to the lower concentration of leaf extracts of *M. sacchariflorus*. However, in the case of *Echinochloa crus-galli* and *Erigeron Canadensis*, even the lower concentration of leaf extracts (100 ppm) significantly inhibited the seed germination. Moreover, a more positive and significant correlation was obtained between the seed germination of the weeds treated with aqueous extracts of *M. sacchariflorus* with chlorogenic acid, rutin, *p*-hydrobenzoic acid, gentisic acid, and ferulic acid (r = 0.924, *p* < 0.01; r = 0.932, *p* < 0.01; r = 0.933, *p* < 0.01; r = 0.997, *p* < 0.01; r = 0.998, *p* < 0.01, respectively). This result indicates that the lower concentrations of *M. sacchariflorus* extracts can stimulate seed germination, while higher concentrations of MS extracts may inhibit germination of weed seeds. *M. sacchariflorus* extracts significantly affected the shoot length as well as root length of weeds (Figure 2B,C). As shown in the present study, the shoot lengths of the weed seedlings were species-specific and were directly proportional to the concentration of allelopathic plant extracts used in the treatment. The shoot length of all of the weeds was inhibited by the *M. sacchariflorus* leaf extracts in a concentration-dependent manner. *M. sacchariflorus* extracts most effectively suppressed the shoot growth of *Bidens frondosa*, *Echinochloa crus-galli*, and *Erigeron canadensis* at a concentration of 10,000 ppm, as compared to the control treatments. The methanolic extracts were least inhibitory to *Amaranthus viridis* seedling growth (shoot length = 4.33 ± 0.47 cm) at same concentration. When compared with the control, the shoot lengths of *Artemisia princeps var. orientalis*, *Commelina communis,* and *Erigeron canadensis* were statistically similar against control treatment, with a slight inhibiting effect.

Gradual decreases in the root length were observed with increased concentrations of *M. sacchariflorus* extracts. The root length of the majority of the weeds was severely reduced by treatment with *M. sacchariflorus* extracts at concentrations of 10,000 ppm. At lower concentrations of *M. sacchariflorus* extracts, the root lengths of *Digitaria ciliaris*, *Oenothera biennis* and *Echinochloa crus-galli* were least affected, as indicated by statistical similarity to the root length of the control treatment. This result indicates that lower concentrations of *M. sacchariflorus* extracts (100 ppm) may not affect the root growth of weed species. In the present study, the root length of weed seedlings was more affected by the leaf extracts than the shoot parts, which may be due to direct exposure of the roots system to the phytotoxins present in the treated extracts. Massive reductions in the seed germination percentage and the shoot and root lengths of the weeds indicates the presence of some phytotoxins in the leaf extracts. The relationship between the germination of the weed seeds and the shoot and root length is shown in Table 3. In particular, at lower concentrations of *M. sacchariflorus* extracts, the shoot length of the weeds showed a higher and more positive correlation with the root length (r = 0.851, r = 760, respectively). A moderate and positive correlation was observed between the rate of seed germination and shoot length in weeds grown with higher concentrations of *M. sacchariflorus* extract, indicating that allelochemicals at higher concentrations may not only inhibit the rate of seed germination but also restrict the growth of the roots and shoots of weeds.

### 2.3. Effects of M. sacchariflorus Mulch on Biomass Production

The fresh weight and dry weight of the weed seedlings were used to assess the allelopathic potential of the plants. In the present study, *M. sacchariflorus* mulch was detrimental for the growth of the tested weed species. The degree of inhibition of fresh weight (FW) and dry weight (DW) of the treated weeds varied in a species-specific manner (Table 4). A significant reduction in the weed population with late emergence was observed in the treated weed seedlings, relative to the control plants. Among the treated weeds, *Commelina communis* was the most sensitive to the allelopathic effects of *Miscanthus* mulch, with the greatest reduction in FW and DW (47.70 g and 4.00 g, respectively). In contrast, *Artemisia princeps var. orientalis, Bidens frondosa,* and *Oenothera biennis* were not detected in the mulched field. The present study suggests that the presence of allelochemicals in the *Miscanthus* mulches exhibited an inhibiting effect in the weed growth and biomass yield. Moreover, a more positive and significant correlation was also obtained between the fresh weight of the treated weeds with the phenolic compounds such as ferulic acid, *m*-coumaric acid, vanillic acid, and vitexin (r = 0.996, *p* < 0.01; r = 0.967, *p* < 0.01; r = 0.999, *p* < 0.01; r = 0.999, *p* < 0.01; respectively) (Table 5).

### 2.4. Effect of M. sacchariflorus Aqueous Extracts of on Electrolyte Leakage

**Figure 3:**
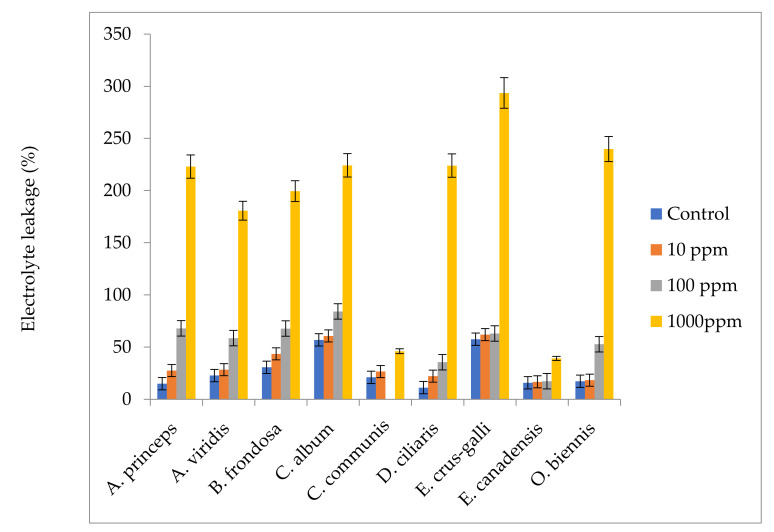
Comparison of electrolyte ion leakage activities of weeds treated with *Miscanthus* extracts. Values are reported as mean ± standard deviation of three parallel experiments.

Aqueous extracts of *M. sacchariflorus* significantly affected the percentage of ion leakage of weeds in a concentration-dependent manner, in comparison to non-treated plants (Figure 3). The largest (over 86%) electrolyte leakage was observed in *Echinochloa crus-galli* treated with 1000 ppm of *M. sacchariflorus* aqueous extracts. Massive increases in electrolyte leakage were also observed in *Oenothera biennis*, *Digitaria ciliaris, Chenopodium album,* and *Artemisia princeps var. orientalis* at the same concentration. The electrolyte leakages of *Digitaria ciliaris* and *Artemisia princeps var. orientalis* were statistically higher even at lower concentrations of aqueous extracts (100 ppm). The least electrolyte leakage was observed in *Commelina communis* and *Erigeron canadensis.* Moreover, a more negative and significant correlation was also obtained between the fresh weight of the treated weeds with the electrolyte Leakage (Table S1).

### 2.5. Effect of M. sacchariflorus Aqueous Extracts on the Photosynthetic Pigments of Weeds

Photosynthetic pigments play an important role in the growth and development of plants and are highly affected by allelochemicals. In the present study, aqueous extracts of *M. sacchariflorus* reduced the concentrations of chlorophyll and carotenoid contents in the treated weeds (Table 6). The most significant reduction in chlorophyll a and chlorophyll b was observed in the seedlings of *Erigeron canadensis* and *Artemisia princeps var. orientalis*, suggesting that the aqueous extracts of *M. sacchariflorus* impaired the photosynthetic pigments. However, *Bidens frondosa* and *Chenopodium album* treated with the same concentration of the extracts showed a smaller reduction (non-significant at *p* = 0.05) of chlorophyll a and b. Relative to the controls, the chlorophyll a contents of *Commelina communis* and *Digitaria ciliaris* were reduced (though not significantly) by the aqueous extracts of *M. sacchariflorus.* As compared to the controls, a slight increase in the chlorophyll b content was observed in *Digitaria ciliaris* and *Commelina communis.* Statistical analysis showed that the aqueous extracts of *Miscanthus sacchariflorus* effectively inhibited carotenoid synthesis in most of the treated weed species. The most significant decrease in the carotenoid content was observed in *Erigeron canadensis* and *Artemisia princeps var. orientalis.* In contrast to the control treatments, the seedlings of *Chenopodium album, Commelina communis,* and *Oenothera biennis* showed increased carotenoid contents. However, the variance analysis showed no significant differences (*p* < 0.05).

### 2.6. Effect of M. sacchariflorus Aqueous Extracts on Antioxidant Enzymatic Activities

#### 2.6.1. SOD Activity

**Figure 4:**
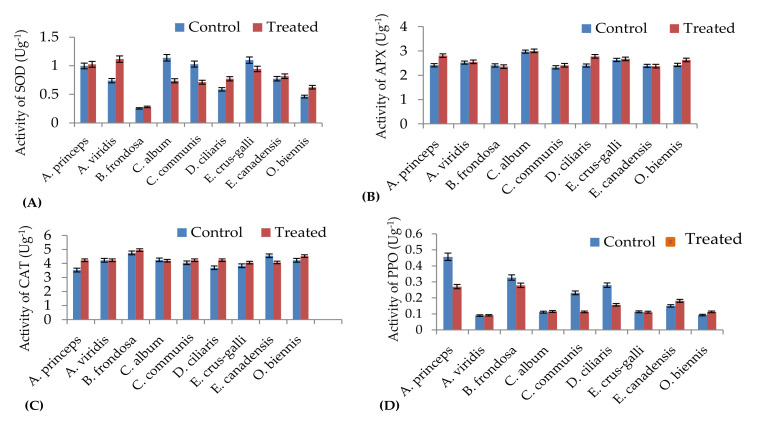
Comparison of antioxidant enzyme activities of weeds treated with *Miscanthus* extracts. (**A**), Superoxide dismutase (SOD); (**B**), Ascorbate peroxidase (APX); (**C**), Catalase (CAT); (**D**), Polyphenoloxidase (PPO). Values are reported as mean ± standard deviation of three parallel experiments.

The influence of the *M. sacchariflorus* extracts on antioxidant enzyme activities is shown in Figure 4A. In the present study, a significant increase in the SOD activity was observed in the majority of the weed species treated with aqueous extracts of *M. sacchariflorus*, compared to the control plants. The greatest increase in SOD activity was observed in *Amaranthus viridis* (1.5 fold compared to the control). However, *Chenopodium album* and *Commelina communis* showed a decreasing trend in SOD activity relative to the control plants. The Pearson correlation coefficient results showed a positive and significant relationship between SOD activity and phenolic compounds such as SOD activity and protocatechuic acid, oreintin, and rutin (r = 0.997, *p* < 0.05; r = 0.999, *p* < 0.05; r = 0.999, *p* < 0.05, respectively) (Table 7), while a strong negative correlation was observed between SOD activity and catechin (r = 0.997, *p* < 0.05).

#### 2.6.2. APX Activity

Changes in the APX activity due to the allelopathic compounds present in the aqueous extracts of *M. sacchariflorus* appeared to vary significantly across the weed species. We observed a considerable increase in APX activity in the majority of treated weed species (Figure 4B). The APX activity was decreased in *Erigeron canadensis* compared to that in the control plants, but this effect was not significant (*p* = 0.05) when compared with the control plants. The Pearson correlation coefficient results showed a more positive and significant relationship between APX activity and phenolic compounds such as gallic acid (r = 0.994, *p* < 0.01), *p*-hydroxybenzoic acid (r = 0.996, *p* < 0.05), and caffeic acid (r = 0.997, *p* < 0.05).

#### 2.6.3. CAT Activity

Enhanced CAT activity was observed in the majority of the weed species treated with the extracts of *M. sacchariflorus* (Figure 4C). The greatest increase in CAT activity was observed in *Digitaria ciliaris* plants treated with the aqueous extracts of *M. sacchariflorus*. Altered APX activities were found in *Amaranthus viridis, Chenopodium album,* and *Erigeron canadensis* seedlings, indicating that the allelochemicals present in the *M. sacchariflorus* extracts altered the antioxidant enzyme activities in the treated weeds species. Correlation analysis revealed a strong, positive, and significant relationship between CAT activity and phenolic compounds such as chlorogenic acid (r = 0.998, *p* < 0.05) and *p*-coumaric acid (r = 0.998, *p* < 0.01).

#### 2.6.4. PPO Activity

Treatment with aqueous extracts of *M. sacchariflorus* yielded a different trend than those of SOD, APX, and CAT. PPO activity decreased in the majority of the treated weeds species, as compared to that of the control plants (Figure 4D). *Artemisia princeps var. orientalis* showed the highest reduction in PPO activity. No significant variance (*p* < 0.05) in the PPO activities was observed in either the treated or control *Amaranthus viridis, Chenopodium album,* and *Echinochloa crus-galli* seedlings. The high correlation between PPO activity and phenolic compounds (ferulic acid and *p*-hydroxybenzoic acid) indicates that antioxidant activity was dependent upon the level of allelopathic compounds. Table 7 summarizes the Pearson’s correlation coefficients between antioxidant enzymes and phenolic compounds present in the aqueous extracts of *M. sacchariflorus*. Strong and negative correlations were found between PPO activity and vitexin, and vanillic acid (r = −0.997, *p* < 0.05; r = −0.998, *p* < 0.05, respectively), indicating that the level of phenolic compounds present in *M. sacchariflorus* extracts could play an important role in the antioxidant capacity of the weeds.

## 3. Discussion

### 3.1. Effect of M. sacchariflorus Leaf Extracts on Seed Germination of Weeds

Previous studies revealed that phytochemicals such as *p*-coumaric acid can affect the inducible water uptake, electrolyte retention capacity, and O_2_ consumption necessary for seed germination [55], indicating that allelochemicals alter or disturb pathways responsible for the synthesis of plant hormones necessary for seed germination. Moreover, these compounds have been shown to reduce the seed germination rate of weeds by inhibiting their respiration [56], effectively altering the permeability of the cell membrane [57,58]. Other studies have documented decreased water uptake [59] with less enzyme activity in germinating weed seeds during allelopathic stress [60]. Phenolic compounds can influence the growth of weeds by affecting the photosynthetic process in the weeds [61]. These compounds can also influence cell differentiation and the levels of metabolites responsible for constructing the cellular machinery necessary for cell elongation [62]. In particular, *p*-hydrobenzoic acid caused significant reduction in the seed germination, seedling length, and dry weight of barnyard grass [18] and inhibited the growth of *Lactuca sativa* [63], *Dactylis glomerata* [64], and *Oryza sativa* [18] in a dose-dependent manner. According to Lehman and Blum [65], *p*-hydrobenzoic acid reduced the hydraulic conductivity and nutrient uptake of weeds, reducing their growth. In another study, higher doses of *P. vulgaris* Mill. leaf extracts, which contained salicylic acid and benzoic acid, prevented the germination of *Hordeum vulgare* [66]. Dikilitas et al. [67] argued that treatment with phenolic compounds such as benzoic acid and *p*-coumarin cause the cytotoxic effect of cell cycle inhibition, indicating that allelochemicals can have different allelopathic effects on different plant species. Reigosa et al. [63] found that a mixture of phenolic compounds suppressed the germination and growth of weed seeds, and found negligible differences in the result using individual compounds. Thus, we infer that the synergistic effects of these compounds could further enhance their natural herbicidal potential for suppressing the growth of weeds.

### 3.2. Effect of M. sacchariflorus Mulch on Biomass Production

Many plant species produce allelochemicals that induce negative effects on the quantum efficiency of photosystem II, reducing the net assimilation of photosynthesis [68,69,70]. In the present study, the dry weight of weeds was lowered by the application of *M. sacchariflorus* mulch. The decrease in dry weight may be due to reduced photosynthetic activity in the seedlings due to the allelochemicals present in the *M. sacchariflorus* extracts. Furthermore, lower root length and shoot length could have contributed to the lower fresh weight and dry weight of the weeds. These findings are corroborated by the report of Chou and Lee [71], who reported the allelopathic effects of aqueous extracts of *Miscanthus transmorrisonensis*. In their study, the phenolic compounds *p*-coumarin, ferullic acid, vanillic acid, protocatechuic acid, *p*-hydroxyphenyl acetic acid, 4-hydroxy coumarin, and phloridzin were reported to have significant phytotoxic effects on rye, grass, lettuce, and Chinese cabbage. In other study, Chou and Lee [71] reported a significant allelopathic effect of *Miscanthus floridulus* on lettuce. Phenolic compounds such as *p*-coumaric, gallic acid, *p*-hydroxybenzoic acid, vanillic acid, vanillin, and ferulic acid were shown to have bioherbicidal properties for natural suppression of weeds in cultivated fields [63]. Therefore, presence of higher *M. sacchariflorus* biomass in the filed would increase the allelochemical concentration and suppressed the weed growth. Further studies are required to identify and isolate the allelochemicals in *M. sacchariflorus* to confirm its suitability for use in natural herbicide applications.

### 3.3. Effect of M. sacchariflorus Aqueous Extracts on Electrolyte Leakage

Electrolyte leakage induced by allelochemicals present in the aqueous extracts of a plant can alter cell membrane integrity and permeability [72,73]. This method is frequently used to quantify cell membrane damage caused by various biotic and abiotic stressors [74,75,76,77,78,79]. Some studies have attributed a significant increase in the electrolyte leakage of plant cells to the phytotoxic chemicals present in plant extracts. In the present study, we observed a high negative correlation between electrolyte leakage and fresh weight (r = −0.920**, *p* < 0.01), suggesting that the allelochemicals effectively inhibited the biomass of the weeds by damaging the permeability of the cell membrane to electrolytes and subsequently causing cell death. Allelochemicals such as flavonoids are known to inhibit the electron transport chain in the mitochondrial membrane [80]. The author observed that phenolic compounds such as cinnamic acid and benzoic acid can cause structural changes in the cell membrane proteins and alter the membrane integrity and permeability. It is, therefore, possible that the treatment of weed seedlings with *M. sacchariflorus* aqueous extracts altered the cell membrane permeability, as indicated by the significant outflow of electrolytes in the tested concentrations.

### 3.4. Effect of M. sacchariflorus Aqueous Extracts on Photosynthetic Pigments

Plant pigments such as chlorophylls a and b and carotenoids play important roles in photosynthesis [81]. Pigment contents (particularly of chlorophyll) are important parameters in studying the mode of action of allelochemicals on plant physiology [82,83]. Changes in these parameters following allelochemical treatment have been reported in several studies [84,85]. The present study revealed that physiological parameters such as chlorophyll a and chlorophyll b were adversely affected by the application of aqueous extracts of *M. sacchariflorus* in the broadleaf and grass weed species. However, chlorophyll a was more affected by the aqueous extracts of *M. sacchariflorus* than chlorophyll b. In the majority of the weed species treated with *M. sacchariflorus* aqueous extracts, the chlorophyll a, chlorophyll b, and carotenoid contents decreased significantly, compared to the untreated plants. These findings are corroborated by the reports of Shao et al. [86], and Sodaeizadeh et al. [87], all of whom observed the degradation of chlorophyll molecules and reduction in photosynthetic rates due to allelochemicals.

Rapid decreases in photosynthetic constituents such as chlorophyll and carotenoids in response to allelochemicals have also been observed in *Cyperus rotundus* treated with sesame leachate [88] and in lettuce treated with *Tignoella foenum-graceum* aqueous extract [89], in accordance with the present results. These findings all indicate that allelochemicals can alter the synthesis of organic compounds, electron donors, and energy (Venkateshwarlu et al. [90], Kaiser et al. [91]. These compounds effectively inhibited protein synthesis in cabbage seedlings [92]. Allelocompounds such as vanillic acid and *p*-hydroxy benzoic acid reduced chlorophyll a content in *Echinochloa crus-galli* [93]. Singh [94] indicated the role of ferulic acid in suppressing the chlorophyll contents of tomato seedlings. Based on these results, we assume that the capacity of our tested weed species to accumulate photosynthetic pigments was also affected by the allelochemicals present in the *M. sacchariflorus* extracts. However, the exact mechanism underlying this effect is unknown.

### 3.5. Effect of M. sacchariflorus Aqueous Extracts on Antioxidant Enzymatic Activities

In the present study, aqueous extracts of *M. sacchariflorus* enhanced the activities of antioxidant enzymes in the majority of the treated weed seedlings. Increases in the SOD activity may be due to the increased production of superoxide radicals, as a result of *de novo* synthesis of proteins, which enhance the transcription of SOD genes [95,96]. CAT and APX, primarily located in the peroxisomes and cytosol of the cells, respectively, are responsible for the efficient scavenging of H_2_O_2_ generated during photorespiration and β-oxidation of fatty acids [97,98]. Other studies have indicated the antioxidant properties of phenolic compounds, but higher concentrations of these compounds may inhibit photosynthetic and antioxidant enzyme activities due to higher ROS generation and cellular damage [99]. Thus, in the present study, it appears that the aqueous extracts of *M. sacchariflorus* caused excessive generation of ROS in the weeds, which activated the activity of antioxidant enzymes to scavenge the ROS. Several previous studies have reported strong allelochemical stress in weed seedlings and the subsequent increase in the antioxidant enzyme activity [100,101,102]. We observed a positive correlation between CAT activity in weeds and the phenolic compounds of aqueous extracts. According to Tripathi et al. [103] and Weir et al. [104], allelochemicals present in plant extracts cause oxidative stress in treated plants, activating the antioxidant mechanisms in the treated plants as an adaptive measure. Thus, it is possible that the phenolic compounds of *M. sacchariflorus* altered the redox balance by changing the physiology of the weed seedlings. In other studies, gallic and caffeic acid have been shown to generate ROS in cells [105,106]. These reactive oxygen species effectively inhibit the activity antioxidant enzymes (including CAT and APX), causing oxidative stress in the cell [107]. Regeneration of ROS may surge as a result of this. To counter the surging phenomenon, antioxidant enzymes may have played a protective role against ROS and phenols at the time of seed germination and seedling growth.

PPO, located in the thylakoid membrane, is known for its important role in photosynthesis [108] and in the oxidation of phenolic compounds into quinones, causing the oxidative browning of plants [109]. We found that the activity of PPO decreased in the majority of the tested weed seedlings. Moreover, in this study, we also observed an inverse correlation between PPO activity and phenolic compounds, including ferulic acid, vitexin, and vanillic acid. This result is corroborated by the report of Tadic et al. [110], in which reductions in the PPO activities in lettuce were attributed to the toxic effects of the treated polyphenols. Thus, our findings indicate that the allelochemicals present in *M. sacchariflorus* could be toxic to weed seedlings and could effectively inhibit the activity of PPO, which is useful in the oxidization and degradation of phenolic compounds, which in turn, can accumulate in treated weeds. Further detailed investigations, including the analysis of the molecular mechanism underlying the activity of antioxidant enzymes through studying gene expression levels, are required to prove these hypotheses.

### 3.6. Conclusions

The present investigation revealed that both mulch and aqueous extracts of MS have a significant influence on the germination and growth of the seedlings of weeds that commonly grow on farmlands. The levels of photosynthetic pigments (chlorophylls and carotenoids) were affected by treatment with plant residues and antioxidant enzymes (SOD, APX, CAT, and PPO). Conversely, the activities of SOD, APX, CAT, and PPO were altered due to the phytotoxic effect of the plant extracts. However, the mechanism by which the plant extracts inhibited or enhanced different physiological parameters and traits of the weed seedlings has not yet been identified and requires further investigation. It is noteworthy that the aqueous extracts of *Miscanthus* may be suitable for application as bio herbicides to control the growth of weeds in cultivated fields.

## 4. Materials and Methods

### 4.1. Chemicals

Analytical standards used in this study were of analytical grade. All analytical standards were purchased from Sigma Chemical Co. (St. Louis, MO, USA). The methanol used for the extraction process was purchased from J.T. Baker (Phillipsburg, NJ, USA).

### 4.2. Plant Material

*M. sacchariflorus* used in this study was cultivated in the experimental fields of Kangwon National University, at Chuncheon, Kangwon-Do, South Korea (37°56′09.96″ N; 127°46′55.21″ E) at an average altitude of 100 m. All of the experimental plots were arranged in a completely randomized block design. The experimental plots consisted of ten longitudinal rows separated by 1.5 m. Each plot consisted of 1.5 m^2^, with one *M. sacchariflorus* plant placed in each plot. The temperature of the cultivated field ranged from −27.9 °C (January) to 39.5 °C (August). The soil of the experimental field had a sandy loam texture. The *M. sacchariflorus* experimental field was irrigated during the initial year of establishment on a regular basis. The research field was located in a temperate monsoon climate, with a wet and humid summer. *M. sacchariflorus* plants were harvested at maturity from the cultivated field and chopped into small pieces (about 4 cm) with an electric cutter and dried at room temperature for 48 h. The dried, chopped pieces of *M. sacchariflorus* were applied to each plot after one week of cultivation. Absence of *M. sacchariflorus* mulch was used as a control.

### 4.3. Effect of M. sacchariflorus Mulch on the Biomass of Weeds

The fresh weight and dry weight of the weeds were measured at 70 days after preparation of the experimental field. Weeds grown in the experimental field in both the treated and control plots were collected from 1-m^2^ quadrats from each plot. The fresh weight of the weeds was recorded immediately after uprooting the weeds, with a digital balance. Dry weight of the weeds was recorded after drying in an oven at 70 °C for 48 h.

### 4.4. Preparation of Plant Extracts

To prepare the aqueous extracts, the leaves of *M. sacchariflorus* were collected from field at maturity. The sample was dried at room temperature for 48 h. The dried sample was ground in a blender. The powder sample (500 g) was mixed with one liter distilled water and the homogenate was kept in room temperature (25 °C) for 24 h. The mixture was filtered using Buchner funnel and filter paper to separate the debris. The obtained aqueous extracts were used for assessing allelopathic effects on weeds.

Allelochemical compounds present in the *M. sacchariflorus* extracts was quantified using an LC-MS/MS system. The fresh leaf samples of *M. sacchariflorus* were collected from the growing field and dried at 25 °C for 24 h, then ground into powder. Dried plant samples were weighed (1 g) and dissolved in 20 mL of 80% methanol for two days at room temperature (25 °C). The extract solutions were filtered through No. 1 Whatman filters and the filtrate was gathered, following which the methanol was evaporated under reduced pressure using a rotary evaporator at 41 °C (Eyela, SB-1300, Shanghai Eyela Co. Ltd. Shanghai, China). The obtained crude methanolic extracts were stored at −20 °C for further use. On the experiment day, the plant extracts were re-dissolved in 80% HPLC graded methanol (0.01 mL).

### 4.5. Effect of M. sacchariflorus Extracts and Allelopathic Compounds on Seed Germination

Seeds of the nine most prominent weeds found in the *M. sacchariflorus* field were collected in November 2018 and 2019 from the experimental fields. The harvested seeds were cleaned and separated from their pods, air-dried at room temperature for a week, and then stored in darkness at 4 °C until further use. Healthy seeds of uniform size were selected and soaked for 24 h in distilled water to remove dirt and germination-inhibiting compounds from the surface of the seeds. Initially, 5 mL of *M. sacchariflorus* methanolic extracts (100 ppm to 1000 ppm) was placed in a sterilized 9 cm diameter petri dishes lined with a double layer of filter papers (No. 1 Whatman) and evaporated to dryness for 12 h at 26 °C. After evaporation, 3 mL of distilled water were added onto the filter paper. Twenty seeds from each weed species were placed on filter papers. Sterile deionized water was used as a control. Three replicates of each treatment were performed and all treatments were maintained at 25 ± 1 °C for 40 days in a growth chamber with an 8:16 light and dark cycle. The effects of the methanolic extracts of *M. sacchariflorus* on weed seed germination and seedling growth (shoot length, root length) were measured after one week of incubation.

### 4.6. Estimation of Phenolic Compounds by Liquid Chromatography-Mass Spectrometry/Mass Spectrometry (LC-MS/MS)

The concentration and composition of phenolic compounds were identified in *M. sacchariflorus* using a liquid chromatography-mass spectrometry/mass spectrometry (LC-MS/MS) system, as described previously [111]. Pumps (Agilent 1200, Agilent Technologies, Palo Alto, CA, USA) and an autosampler (Agilent 1100 series, Agilent Technologies, Palo Alto, CA, USA) were coupled to an API 2000 series mass spectrometer (Applied Biosystems, ON, Canada) integrated to the LC system. The chromatographic separation of phenolic compounds was performed using a C18 column (4.6 × 250 mm, 5 μm). The mass spectrometer was used in negative ion mode. The following parameters were used to determine the phenolic compounds present in the samples: nebulizer gas pressure, drying gas pressure, collision gas pressure, and curtain gas pressure set at 40, 70, 2, and 20 psi, respectively; drying gas temperature and capillary voltage were set at 350 °C and 4.5 kV, respectively. The mobile phase comprised 0.1% formic acid (HCOOH) (*v/v*) in water (solution A) and acetonitrile (C_2_H_3_N) in water (solution B). The following mobile phase gradient elution programs were performed to efficiently separate the compounds: 10–40% B for 0–10 min; 40–50% B for 10–20 min; 50–100% B for 20–25 min; 100–10% B for 25–26 min; and 10% B for 26–30 min. During the experiment, the temperature of the column was set at 25 °C. The sample injection volume was 10 µL. The mobile phase was eluted at a constant flow rate of 0.7 mL min^−1^. Mass-spectrometry data were recorded in multiple reactions monitoring (MRM) mode. Different mass spectrometric parameters such as entrance potential (EP), collision energy (CE), declustering potential (DP), cell entrance potential (CEP), and collision cell exit potential (CXP) were determined for each MRM transition that was monitored. Analysis of allelochemicals for each sample was performed in triplicate.

### 4.7. Effect of M. sacchariflorus Extracts on Photosynthetic Pigment Contents

To extract chlorophyll a, chlorophyll b, and carotenoids, 0.5 g fresh leaf samples of weeds from the treated and control plots were taken and homogenized with 10 mL of 80% acetone. Homogenized samples were centrifuged for 4000 rpm for 10 min at 4 °C. The supernatants were separated from the mixture and collected in a cuvette for further use. To measure the chlorophyll a, chlorophyll b, and carotenoid contents, the spectrophotometric method proposed by Lichtentaler and Wellburn [112] was employed using a Shimadju UV-VS spectrophotometer (UV-1800, Shimadzu, Kyoto, Japan). The concentrations of photosynthetic pigments present in the extracts were estimated using the following equations:

Chlorophyll-a = 12.25A_663.2_ − 279A_646.8_ (1)

Chlorophyll-b = 21.5A_646.8_ − 5.1A_663.2_ (2)

Carotenoids = (1000A_470_ − 1.82Chl_a_ − 85.02Chl_b_)/198 (3)

### 4.8. Effect of M. sacchariflorus Extracts on Electrolyte Leakage

Leaf discs (2 g) from fresh young leaves were cut into 2 cm segments and rinsed in deionized water, then placed in petri dishes containing 20 mL of distilled water for 24 h at 25 °C. The samples were then washed in an orbital shaker (Orbit Shaker, Lab-Line, Dubuque, USA) at room temperature. The initial electrical conductivity (EC0) of the samples was measured at 15 min intervals using a digital conductometer (Digimed DM-3, Digicron, Sao Paulo, Brazil) [113]. The petri dishes containing cut samples were placed in a boiling water bath for 30 min and then cooled at 25 °C. The electrical conductivity of the cooled samples was then measured (ECf). Electrolyte leakage (EL) was determined according to the following equation: EL = (ECf−EC0), where ECf is the electrical conductivity at the final time and EC0 is the initial electrical conductivity.

### 4.9. Antioxidant Enzyme Assay

For superoxide dismutase (SOD) activity, fresh seedling leaves (1 g) of treated and non-treated (control) weeds were homogenized in a mortar and pestle using liquid nitrogen in 5 mL of extraction buffer (20 mM Tris-HCl in 1% polyvinylpyrrolidone, pH 7.4) following the method described by Beauchamp and Fridovich [114]. The extracted solution was filtered to remove the debris, and the homogenate was centrifuged at 10,000 *g* for 30 min at 4 °C. The supernatant was separated from the solution and then used for antioxidant assays. An initial reaction solution was prepared by mixing 50 mM phosphate buffer (pH 7.8), 75 μM nitroblue tetrazolium, 13 mM methionine, 100 nM EDTA, 2 μM riboflavin, and dd H_2_O. After two minutes, 50 μL of the enzyme extract was added. The absorbance value of the reaction mixture was then recorded at 560 nm. One unit of SOD activity (U) was defined as the quantity of enzyme required for 50% inhibition of the initial reaction rate.

Ascorbate peroxidase (APX) activity was estimated as described by Nakano and Asada [115]. Initially, 50 mM potassium phosphate (pH 7.0), 0.5 mM ascorbic acid (C_6_H_8_O_6_), 2% hydrogen peroxide (H_2_O_2_), 0.2 mM Ethylenediaminetetraacetic acid (EDTA), and 0.1 mL enzyme extract were mixed to a final volume of 3 mL. The absorbance value of the mixture was measured via Shimadju UV-VS spectrophotometer (UV-1800, Shimadzu, Kyoto, Japan) at 290 nm. The quantity of ascorbate oxidized was calculated using the extinction coefficient (2.8 mM^−1^ cm^−1^). APX was defined as 1 mmol mL^−1^ per min at 25 °C cm^−1^). One unit of ascorbate oxidized as 1 mmol mL^−1^ ascorbate oxidized per min at 25 °C.

Catalase (CAT) assay indicates the variation in the quantity of H_2_O_2_ due to the presence of CAT in the test samples, indicating the potency of the activator/inhibitors under assessment. The catalase (CAT) assay was conducted following the methods described by Aebi [116]. Initially, various extracts with different concentrations were added to the 4 uL of plant extracts in 67 mM sodium potassium phosphate at pH 7.4. The mixture was further incubated for 10 min at 37 °C. Then, 1.2 mL of H_2_O_2_ (0.6%) was added to the mixture and the change in absorbance was assessed at 240 nm for 2 min. Sodium azide was used as a positive control.

Polyphenol oxidase activity (PPO) was determined following the method described by Saeidian [117]. The reaction mixture consisted of 100 mM phosphate buffer (pH 7.0), 1 mL (0.1 M) catechol, and 0.5 mL of enzyme extracts mixed to a final volume of 3 mL. The mixture was incubated at room temperature for five minutes. The PPO activity assay was carried out using a Shimadju UV-VS spectrophotometer (UV-1800, Shimadzu, Kyoto, Japan) at 420 nm.

### 4.10. Statistical Analysis

The data shown represent the mean ± SD. The data were statistically evaluated using analysis of variance (ANOVA) and significant differences between the means were assessed using Duncan’s multiple range tests at significance levels of *p* < 0.05 and *p* < 0.01. Interrelationships among seed germination and growth, pigment contents, phenolic compounds, electrolyte ion leakage, and antioxidant enzyme activities were determined by Pearson’s correlation coefficient using SPSS version 20 (SPSS, 2011).

## Figures and Tables

**Table 1:** LC-MS/MS parameters for the quantitative analysis of 22 phenolic compound standards.

Compounds	Retention Time	Q1 (*m/z*)^a^	Q3(*m/z*)^b^	DP (V)^c^	EP (V)^d^	CEP (V)^e^	CE (eV)^f^	CXP (V)^g^
L-Phenylalanine	5.80	163.888	146.8	−41.000	−10.500	−12.000	−18.000	−10.000
Gallic acid	5.92	169.056	125.000	−26.000	−10.000	−8.000	−20.000	−6.000
Homogentisic acid	7.10	166.920	122.900	−21.000	−7.000	−14.000	−14.000	−28.000
Protocatechuic acid	8.04	152.896	108.900	−16.000	−9.000	−10.000	−22.000	−6.000
Chlorogenic acid	8.30	352.846	191.000	−21.000	−7.000	−32.000	−30.000	−10.000
Orientin	9.69	447.092	327.000	−61.000	−11.000	−20.000	−22.000	−54.000
Rutin	9.98	609.002	299.700	−91.000	−10.500	−34.000	−52.000	−14.000
*p*-Hydroxybenzoic acid	10.02	136.885	92.900	−16.000	−8.500	−12.000	−24.000	−6.000
Caffeic acid	10.10	178.868	134.800	−16.000	−8.000	−14.000	−22.000	−6.000
Vitexin	10.36	430.826	310.800	−56.000	−9.000	−28.000	−22.000	−50.000
Vanillic acid	10.46	166.883	151.900	−11.000	−6.500	−10.000	−18.000	−32.000
Gentisic acid	10.63	152.871	107.900	−16.000	−8.500	−6.000	−28.000	−22.000
2**,**4**-**Dihydroxybenzoic acid	11.42	152.900	108.900	−21.000	−4.500	−10.000	−18.000	−6.000
*p*-Coumaric acid	12.08	162.866	118.900	−11.000	−7.000	−14.000	−20.000	−6.000
Ferulic acid	12.44	192.881	133.900	−11.000	−10.000	−12.000	−22.000	−8.000
*m*-Coumaric acid	12.58	162.886	118.900	−21.000	−9.000	−8.000	−22.000	−4.000
Veratric acid	12.96	180.991	136.900	−16.000	−7.500	−12.000	−18.000	−8.000
Luteolin	15.77	284.966	133.200	−66.000	−10.000	−16.000	−46.000	−30.000
Quercetin	16.11	300.884	150.800	−31.000	−10.500	−18.000	−28.000	−32.000
Salicylic aci	16.58	136.874	92.900	−16.000	−7.500	−12.000	−22.000	−6.000
Apigenin	18.50	268.948	117.000	−46.000	−10.000	−20.000	−56.000	−8.000

^a^ Precursor ion (Q1, m/z); ^b^ fragment ion (Q3, m/z); ^c^ DP: declustering potential; ^d^ EP: entrance potential; ^e^ CE: collision energy; ^f^ CEP: cell entrance potential; ^g^ CXP: collision cell exit potential.

**Table 2:** Allelopathic compound contents of the methanolic extracts of *Miscanthus* leaves identified and quantified by LC-MS/MS.

Name of Phenolic Compounds	Coefficient of Determination (r^2^)	Limit of Quantification LOQ (μg mL^−1^)	CAS Number	Purity (%)	Concentration of Phenolic Compounds (µg g^−1^) **
L-Phenylalanine	1.00	1.786	673-06-3	99.0	< LOQ
Gallic acid	0.99	0.025	149-91-7	97.0	4.49 ± 0.44^h^
Homogentisic acid	0.99	0.037	451-13-8	97.0	< LOQ
Protocatechuic acid	0.99	0.005	99-50-3	≥ 98.0	56.80 ± 4.60^s^
Chlorogenic acid	1.00	0.030	327-97-9	98.0	296.00 ± 1.00^dd^
Orientin	0.99	0.781	28608-75-5	≥ 97.0	512.71 ± 3.09^u^
Rutin	0.99	0.114	207671-50-9	≥ 94.0	27.53 ± 0.49^j^
*p*-Hydroxybenzoic acid	1.00	0.022	99-96-7	≥ 99.0	14.07 ± 0.25^b^
Caffeic acid	0.99	0.007	331-39-5	≥ 98.0	11.96 ± 0.23^m^
Vitexin	0.99	0.147	3681-93-4	95.0	96.10 ± 0.31^z^
Vanillic acid	1.00	1.667	121-34-6	≥ 97.0	ND
Gentisic acid	1.00	0.011	4955-90-2	≥ 99.5	3.38 ± 0.09^e^ *
2**,**4**-**Dihydroxybenzoic acid	0.99	0.010	89-86-1	≥ 97.0	11.89 ± 1.34^q^
*p*-Coumaric acid	0.99	0.006	501-98-4	≥ 98.0	58.07 ± 3.15^n^
Ferulic acid	0.99	0.111	537-98-4	≥ 99.0	42.93 ± 2.35^o^
*m*-Coumaric acid	0.99	0.010	588-30-7	≥ 95.0	5.27 ± 0.21^de^
Veratric acid	1.00	2.632	93-07-2	≥ 99.0	384.38 ± 5.14^k^
Luteolin	1.00	0.068	491-70-3	≥ 98.0	409.33 ± 3.32^cc^
Quercetin	1.00	0.042	117-39-5	≥ 95.0	5.19 ± 0.17^c^
Salicylic acid	0.99	0.005	69-72-7	≥ 99.0	1.24 ± 0.22^d^
Apigenin	0.99	0.026	520-36-5	≥ 95.0	10.79 ± 0.23^x^

**The values of individual compounds were within the mean ± standard deviation (n = 3). Data having the same letter in a column were not significantly differentiated by Duncan’s multiple comparison test (*p* < 0.05). *ND, not detected.

**Table 3:** Pearson correlation coefficients (r) between seed germination (%), shoot length (cm), and root length (cm) at different concentrations of *M. sacchariflorus* extracts.

	^1^G100	G1000	G10000	S100	S1000	S10000	R100	R1000	R10000
G100	1								
G1000	0.941 **	1							
G10000	0.811 **	0.629	1						
S100	0.014	−0.057	−0.257	1					
S1000	0.086	0.029	−0.206	0.968 **	1				
S10000	0.692 *	0.723 *	0.447	−0.150	−0.118	1			
R100	−0.014	−0.161	−0.185	0.851 **	0.760 *	0.012	1		
R1000	0.195	0.059	0.026	0.569	0.501	0.412	0.862 **	1	
R10000	0.367	0.316	0.292	−0.185	−0.235	0.766 *	0.240	0.678 *	1

** Correlation is significant at the 0.01 level (2-tailed). * Correlation is significant at the 0.05 level (2-tailed).^1^G100, S100, R100; seed germination rate, shoot length, and root length of weeds treated with 100 ppm of *M. sacchariflorus* extracts. G1000, S1000, R1000; seed germination rate, shoot length, and root length of weeds treated with 1000 ppm of *M. sacchariflorus* extracts. G10000, S10000, R10000; seed germination rate, shoot length, and root length of weeds treated with 10,000 ppm of *M. sacchariflorus* extracts.

**Table 4:** Allelopathic effects of *M. sacchariflorus* extract on fresh weight and dry weight of broad-leaved weeds and grass weeds.

Plant Species	Control **		Treated	
	Fresh Weight	Dry Weight	Fresh Weight	Dry Weight
*A* *. princeps (Pamp.) Hara*	141.50 ± 2.00	25.00 ± 1.50	*ND	ND
*A. viridis* L.	121.50 ± 3.00	24.50 ± 1.00	25.90 ± 2.50	4.50 ± 0.50
*B. frondosa* L.	51.05 ± 2.00	8.00 ± 1.00	ND	ND
*C. album* L.	17.33 ± 2.60	4.53 ± 0.70	5.00 ± 1.00	0.28 ± 0.10
*C. communis* L.	192.00 ± 4.00	19.00 ± 2.00	47.70 ± 2.10	4.00 ± 0.67
*D. ciliaris* (Retz.) Koel.	52.67 ± 3.47	26.09 ± 8.07	1.00 ± 0.14	0.33 ± 0.11
*E.crus-galli* L.	109.50 ± 2.50	17.00 ± 0.90	2.50 ± 1.47	1.00 ± 0.29
*E.canadensis* L.	68.50 ± 7.80	23.29 ± 2.50	74.50 ± 1.50	15.00 ± 0.80
*O. biennis* L.	41.50 ± 3.50	8.86 ± 1.50	ND	ND

*ND, not detected; ** Values are reported as mean ± standard deviation of three parallel experiments.

**Table 5:** Pearson correlation coefficients (r) between seed germination, shoot length, root length, electrolyte leakage, fresh weight, and phenolic compounds of *M. sacchariflorus*.

	^1^GER	SL	RL	EL	FW	GA	PR	CH	ORE	RU	*p*-HY	CA	VIT	VA	2,4-DHBA	*p*-C	FE	*m*-C	VE	LUT	QU	SA	API
GER	1	0.447	0.292	0.195	−0.204	0.258	0.860	−0.719	0.848	0.841	0.225	0.254	0.932	0.933	0.788	−0.752	0.997 *	0.912	−0.647	0.952	0.998 *	−0.017	−0.156
SL	0.447	1	0.766 *	−0.011	0.005	0.147	0.797	−0.636	0.783	0.774	0.114	0.144	0.967	0.967	0.852	−0.672	0.999 *	0.952	−0.557	0.911	0.999 *	0.096	−0.044
RL	0.292	0.766 *	1	0.135	−0.170	0.015	0.710	−0.529	0.693	0.684	−0.019	0.011	0.992	0.992	0.914	−0.569	0.986	0.984	−0.442	0.849	0.983	0.227	0.089
EL	0.195	−0.011	0.135	1	−0.920 **	0.615	−0.111	−0.121	−0.088	−0.074	0.642	0.618	−0.851	−0.850	−0.966	−0.073	−0.663	−0.877	−0.218	−0.329	−0.650	−0.788	−0.694
FW	−0.204	0.005	−0.170	−0.920 **	1	−0.074	0.644	−0.450	0.626	0.615	−0.108	−0.078	0.999 *	0.999 *	0.947	−0.493	0.967	0.996	−0.360	0.798	0.963	0.313	0.178

* Correlation is significant at the 0.05 level (2-tailed). ** Correlation is significant at the 0.01 level (2-tailed). ^1^GER: Germination, SL: Shoot length, RL: Root length, EL: Electrolyte leakage, FW: Fresh weight, GA: Gallic acid, PR: Protocatechuic acid, CH: Chlorogenic acid, ORE: Orientin, RU: Rutin, *p*-HY: *p*-Hydroxybenzoic acid, CA: Caffeic acid, VIT: Vitexin, GE: Gentisic acid, VA: Vanillic acid, 2,4-DHBA: 2,4-Dihydroxybenzoic acid, *p*-C: *p*-Coumaric acid, FE: Ferulic acid, *m*-C: m-Coumaric acid, VE: Veratric acid, LUT: Luteolin, QU: Quercetin, SA: Salicylic acid, API: Apigenin.

**Table 6:** Effect of aqueous extract of *Miscanthus* leaves on photosynthetic pigments of weeds.

Plants Species	Chlorophyll a		Chlorophyll b		Carotenoid *		Ratio a/b	
	**Control**	**Treated**	**Control**	**Treated**	**Control**	**Treated**	**Control**	**Treated**
*A. princeps* *(Pamp.) Hara*	2.94 ± 0.01	2.18 ± 0.01	1.46 ± 0.01	0.91 ± 0.01	2.34 ± 0.04	1.96 ± 0.01	2.019	2.41
*A. viridis L.*	3.34 ± 0.02	2.91 ± 0.02	2.03 ± 0.01	1.33 ± 0.01	3.72 ± 0.02	2.76 ± 0.03	1.64	2.19
*B. frondosa L.*	3.25 ± 0.01	3.24 ± 0.01	3.32 ± 0.01	3.21 ± 0.01	3.96 ± 0.03	3.98 ± 0.03	0.98	1.01
*C. album L.*	3.19 ± 0.01	3.17 ± 0.01	1.87 ± 0.01	1.86 ± 0.01	3.81 ± 0.03	3.89 ± 0.08	1.71	1.72
*C. communis L.*	3.07 ± 0.01	3.24 ± 0.01	1.44 ± 0.01	2.51 ± 0.01	2.93 ± 0.01	3.98 ± 0.01	2.15	1.29
*D. ciliaris* (Retz.) Koel.	3.29 ± 0.11	3.39 ± 0.01	2.95 ± 0.01	3.54 ± 0.29	3.99 ± 0.01	3.94 ± 0.05	1.12	0.96
*E. crus-galli L.*	3.17 ± 0.01	2.92 ± 0.01	1.77 ± 0.01	1.21 ± 0.01	3.73 ± 0.03	2.65 ± 0.02	1.79	2.41
*E. Canadensis L.*	2.67 ± 0.01	1.96 ± 0.01	1.13 ± 0.01	0.79 ± 0.01	2.46 ± 0.01	1.76 ± 0.01	2.35	2.48
*O. biennis L.*	3.03 ± 0.01	3.18 ± 0.01	1.45 ± 0.01	2.02 ± 0.01	3.02 ± 0.01	3.71 ± 0.02	2.08	1.58

* Values are reported as mean ± standard deviation of three parallel experiment.

**Table 7:** Pearson correlation coefficients (r) between antioxidant enzymes and phenolic compounds of *M. sacchariflorus* extracts.

	SOD	APX	CAT	PPO	GA	PR	CH	ORE	RU	*p*-HY	CA	VIT	VA	2,4-DHBA	*p*-C	FE	*m*-C	VE	LUT	QU	SA	API
SOD	1	0.346	−0.810 **	−0.359	0.766	0.997 *	−0.988	0.999 *	0.999 *	0.743	0.763	0.555	0.557	0.292	−0.994	0.772	0.512	−0.967	0.955	0.783	−0.587	−0.695
APX	0.346	1	−0.351	−0.173	0.994 **	0.712	−0.854	0.728	0.737	0.996 *	0.997 **	−0.115	−0.113	−0.397	−0.828	0.177	−0.166	−0.901	0.537	0.194	−0.972	−0.995
CAT	−0.810 **	−0.351	1	0.499	−0.826	−0.985	0.998 *	−0.988	−0.990	−0.807	−0.824	−0.469	−0.471	−0.194	0.998 **	−0.704	−0.423	0.988	−0.921	−0.716	0.666	0.763
PPO	−0.359	−0.173	0.499	1	0.038	−0.671	0.483	−0.654	−0.644	0.072	0.042	−0.997 *	−0.998 *	−0.934	0.524	−0.976	−0.992	0.394	−0.820	−0.972	−0.278	−.0141

** Correlation is significant at the 0.01 level (2-tailed); * Correlation is significant at the 0.05 level (2-tailed). SOD Superoxide dismutase, APX Ascorbate peroxidase, CAT Catalase; PPO Polyphenoloxidase, GA: Gallic acid, PR: Protocatechuic acid, CH: Chlorogenic acid, ORE: Orientin, RU: Rutin, *p*-HY: *p*-Hydroxybenzoic acid, CA: Caffeic acid, VIT: Vitexin, GE: Gentisic acid, VA: Vanillic acid, 2,4-DHBA: 2,4-Dihydroxybenzoic acid, *p*-C: *p*-Coumaric acid, FE: Ferulic acid, *m*-C: *m*-Coumaric acid, VE: Veratric acid, LUT: Luteolin, QU: Quercetin, SA: Salicylic acid, API: Apigenin.

## Supplementary Materials

The following are available online at https://www.mdpi.com/2223-7747/9/10/1313/s1. Table S1: Pearson correlation coefficients (r) between fresh weight, dry weight, seed germination (%), shoot length (cm), and root length (cm).
