# Supplementary material for: Screening of Allelochemicals in Miscanthus sacchariflorus Extracts and Assessment of Their Effects on Germination and Seedling Growth of Common Weeds
Source: Plants (Basel). 2020 Oct 5;9(10):1313. doi: 10.3390/plants9101313 (PMC7600465; doi:10.3390/plants9101313)
Supplement: Supplementary file 1 [file plants-09-01313-s001.pdf]

Table S1. Pearson correlation coefficients (r) between fresh weight, dry weight, seed germination (%), shoot length (cm), root length (cm, electrolyte leakage and photosynthetic pigments.

	DW	FW	GER	ST	RT	EL	CHL a	CHL b	CAR	CHL a/b
DW	1									
FW	0.939**	1								
GER	−0.220	−0.204	1							
ST	−0.078	0.005	0.447	1						
RT	−0.228	−0.170	0.292	0.766*	1					
EL	−0.778*	−0.920**	0.195	−0.011	0.135	1				
CHL a	−0.643	−0.491	0.217	0.230	0.535	0.328	1			
CHL b	−0.457	−0.346	−0.064	0.013	0.435	0.080	0.809**	1		
CAR	−0.564	−0.387	0.180	0.083	0.417	0.146	0.945**	0.865**	1	
CHL a/b	0.443	0.298	−0.035	−0.014	−0.394	0.000	−0.811**	−0.981**	−0.909**	1

** Correlation is significant at the 0.01 level (2-tailed). * Correlation is significant at the 0.05 level (2-tailed). [1]DW; Dry weight, FW; Fresh weight, GER; seed germination rate, ST; Shoot length, RT, Root length, EL; Electrolyte leakage, CHL a; Chlorophyll a, CHL b; Chlorophyll b, CAR; Carotenoid, CHL a/b; Ratio of Chlorophyll a/ b.
